# GuideLiner Catheter Use for Percutaneous Intervention Involving Anomalous Origin of a Single Coronary Trunk Arising from the Ascending Aorta

**DOI:** 10.1155/2016/8790347

**Published:** 2016-07-27

**Authors:** Mitsunari Matsumoto, Yusuke Tamanaha, Yoshimasa Tsurumaki, Tomohiro Nakamura

**Affiliations:** Department of Internal Medicine, Saitama Citizens Medical Center, Saitama 331-0054, Japan

## Abstract

Cases in which an anomalous single coronary trunk arises from the ascending aorta are extremely rare. In percutaneous coronary intervention for the lesion of a coronary artery anomaly, several problems may occur, including selection of a guide catheter, insufficient backup force, and difficulties of stent delivery. The GuideLiner catheter, which is a coaxial guide extension having the advantage of rapid exchange, facilitates coronary intervention for complex lesions. We report a case of angina having a lesion in the left anterior descending artery of a single coronary trunk arising from the ascending aorta. We successfully performed revascularization by using the GuideLiner catheter.

## 1. Introduction

Coronary artery anomalies are a diverse group of congenital disorders with many variations. Coronary artery anomalies of anomalous origin are uncommon but are occasionally encountered in clinical practice, with an incidence of 0.3–5% [1, 2]. In addition, a single coronary trunk is also rare, with an incidence of 0.12% [3]. We experienced an extremely rare case involving a single coronary trunk arising anomalously from the ascending aorta. This patient's unusual anatomy led to several problems, including selection of the guide catheter, insufficient backup force, and difficulties of stent delivery in percutaneous coronary intervention (PCI). We report a rare case with a single coronary trunk of anomalous origin that was successfully treated using the GuideLiner catheter (Vascular Solutions, MN, USA).

## 2. Case

A 65-year-old male patient was admitted to our hospital complaining of chest oppression during light exertion. The patient's coronary risk factors included hypertension and dyslipidemia. His serum creatinine level was 1.22 mg/dL and estimated glomerular filtration rate was 47 mL/min/1.73 m^2^. An electrocardiogram obtained at rest showed sinus rhythm and no significant ST-T change. Echocardiography showed normal function and normal chamber size. He underwent coronary angiography via the transradial approach to estimate cardiac ischemia.

**Figure 1:**
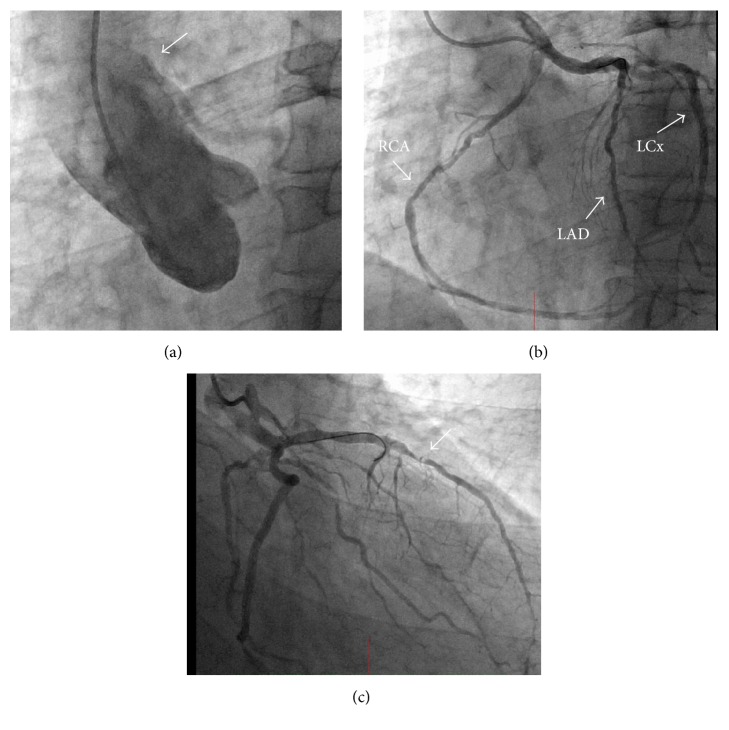
(a) Aortography with a pigtail catheter showing the anomalous origin of the coronary artery from the ascending aorta. (b) Coronary artery angiography with 5-Fr Amplatz Left-1 (AL-1) diagnostic catheter showing the single coronary trunk. (c) Right anterior oblique view showing critical stenosis in the middle segment of the left anterior descending (LAD) artery. A 0.014-inch guide wire was advanced into the LAD in order to stabilize the diagnostic catheter.

Coronary angiography showed no vessel arising from each coronary sinus. Aortography showed that the coronary trunk was originating from the ascending aorta (Figure 1(a)). A 5-Fr diagnostic Amplatz Left-1 (AL-1) catheter was engaged and demonstrated a single coronary trunk (Figure 1(b)). In order to stabilize the diagnostic catheter, a 0.014-inch guide wire was advanced into the septal branch of the left anterior descending (LAD) artery. The middle segment of the LAD was critically stenosed (Figure 1(c)). The lesion in the middle of the LAD was considered the cause of the patient's symptoms; then we planned to perform PCI via the transfemoral approach at a later date.

**Figure 2:**
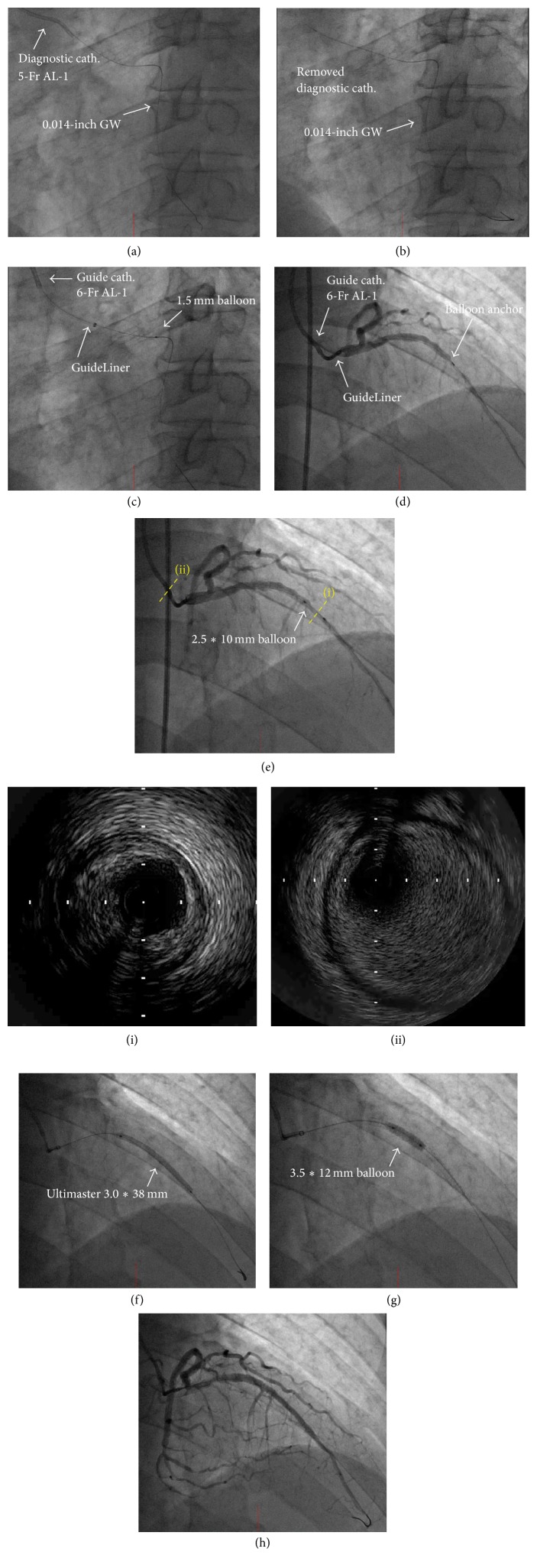
(a) We failed to engage the coronary trunk by using a 6-Fr AL-1 guiding catheter. We then engaged the coronary trunk by using a 5-Fr diagnostic catheter, and a 0.014-inch guide wire was advanced across the LAD lesion. (b) Removing the 5-Fr diagnostic catheter, leaving only the guide wire. (c) A 1.5 mm semicompliance balloon in the lead, along with a mother-child system, including a 6-Fr AL-1 guide catheter and a GuideLiner catheter, was advanced close to the orifice of the coronary trunk. (d) After anchoring it with a 1.5 mm balloon, the GuideLiner catheter was selectively introduced into the left coronary artery. In order to avoid coronary dissection, the contrast medium was gently introduced via manual injection. (e) Angiogram after dilatation with a 2.5 mm semicompliance balloon. Intravascular ultrasound (IVUS) images at the culprit lesion (i) and at the coronary ostium (ii). (f) Deploying a bioresorbable polymer sirolimus-eluting stent (Ultimaster® 3.0*∗*38 mm). (g) Additional dilatation with a noncompliance balloon 3.5*∗*12 mm. (h) Final angiogram showing adequate dilatation of the culprit lesion.

Figure 2 shows the results of the PCI performed on this patient. We failed to engage the anomalous orifice of the coronary trunk by using a 6-Fr AL-1 guiding catheter (Boston Scientific, MN, USA). Therefore, we utilized a 5-Fr diagnostic AL-1 catheter to engage the coronary trunk and a 0.014-inch floppy guide wire (Sion blue®, Asahi Intecc, Aichi, Japan) was advanced into the distal portion of the LAD (Figure 2(a)). After extending the guide wire, we removed the diagnostic catheter and left only the guide wire (Figure 2(b)). A 1.5 mm semicompliance balloon in the lead, along with a mother-child system, including a 6-Fr AL-1 guide catheter and a GuideLiner catheter, was advanced close to the orifice of the coronary trunk (Figure 2(c)). Then, the GuideLiner catheter was selectively inserted into the left coronary artery by anchoring it coaxially with the 1.5 mm balloon (Figure 2(d)). In order to avoid coronary dissection, contrast medium was gently injected using a manual method.After dilatation with a 2.5 mm semicompliance balloon (Figure 2(e)), we have implanted the bioresorbable polymer sirolimus-eluting stent (Ultimaster 3.0*∗*38 mm, TERUMO, Tokyo, Japan) under the intravascular ultrasound guide (Figure 2(f)). The stent was additionally dilated with a noncompliance balloon 3.5*∗*12 mm (Figure 2(g)). A final angiogram showed adequate and favorable dilatation of the culprit lesion (Figure 2(g)). During the procedure, the volume of contrast medium used was 75 mL, and the radiation exposure dose was 0.78 Gy.

## 3. Discussion

Using the GuideLiner catheter, we successfully treated a rare case of angina with a single coronary trunk arising anomalously from the ascending aorta. During PCI for a lesion with coronary anomalies, several problems are encountered, including selection of an appropriate guiding catheter, insufficient backup force, and difficulties in device delivery. We believe that the use of GuideLiner catheter could help to resolve these problems.

The GuideLiner catheter, which has been available in Japan since 2014, is a rapid exchange “mother-child” guide extension that permits deep and selective intubation of the vessel. The conventional “mother-child” system is usually complicated because of its over-the-wire system, and there is sometimes concern about the length of the device such as guide wire, balloon, or stent when using the conventional system. The GuideLiner catheter has three merits for its use: deep seating for additional backup support to facilitate device delivery, coaxial alignment if there is failed engagement of the guiding catheter coaxiality, and reducing contrast volume by selective injection [4–6]. Because the GuideLiner catheter provides backup support, this device is generally utilized for lesions involving calcification or/and coronary tortuosity. In addition, when it is difficult to select an appropriate guiding catheter selection due to an anomalous origin of the coronary artery, the GuideLiner catheter is thought to be effective. In our case, although we failed to engage the AL-1 guiding catheter by the conventional method, the GuideLiner catheter could be selectively introduced to the coronary artery by using a balloon anchor technique. Moreover, in cases involving a single coronary trunk, this device can facilitate subselective visualization of the targeted coronary arteries and therefore has a potential use in minimizing contrast delivery during PCI. The volume of contrast medium during PCI in our case was 75 mL, and follow-up assessment confirmed the absence of contrast-induced nephropathy. Although the GuideLiner catheter has been useful for complex PCI, we should be careful to prevent vessel injury and dissection [7]. Especially in a case with a single coronary trunk, we should use the GuideLiner catheter with extreme caution during the procedure to avoid the coronary dissection and ischemia induced by deep seating. Our practice is to deliver the GuideLiner catheter over an anchoring balloon catheter and to carefully inject the contrast medium manually through the GuideLiner. Finally, we should ensure that there has been no proximal trauma and dissection.

We experienced an extremely rare case with single coronary trunk arising from the ascending aorta. In complex PCI for anomalous coronary arteries, the GuideLiner catheter is useful in facilitating deep seating and for increasing backup force.
